# Impact of the COVID-19 Pandemic on Clinical Findings in Medical Imaging Exams in a Nationwide Israeli Health Organization: Observational Study

**DOI:** 10.2196/42930

**Published:** 2023-05-02

**Authors:** Michal Ozery-Flato, Liat Ein-Dor, Ora Pinchasov, Miel Dabush Kasa, Efrat Hexter, Gabriel Chodick, Michal Rosen-Zvi, Michal Guindy

**Affiliations:** 1 IBM Research - Israel Haifa Israel; 2 Assuta Medical Centers Tel Aviv Israel; 3 Maccabi Healthcare Services Tel Aviv Israel; 4 Sackler Faculty of Medicine Tel Aviv University Tel Aviv Israel; 5 Faculty of Medicine The Hebrew University Jerusalem Israel; 6 Goldman Medical School Ben Gurion University of the Negev Beer Sheva Israel

**Keywords:** COVID-19, COVID, coronavirus, postacute COVID-19 syndrome, PCS, long COVID, pandemic, vaccination, vaccine, health services, utilization, medical imaging, radiology, MRI, CT, Mammography, observational study, electronic health records, EHRs, causal inference, causal effect, inverse probability weighting, IPW, balancing weights, adversarial balancing, machine learning

## Abstract

**Background:**

The outbreak of the COVID-19 pandemic had a major effect on the consumption of health care services. Changes in the use of routine diagnostic exams, increased incidences of postacute COVID-19 syndrome (PCS), and other pandemic-related factors may have influenced detected clinical conditions.

**Objective:**

This study aimed to analyze the impact of COVID-19 on the use of outpatient medical imaging services and clinical findings therein, specifically focusing on the time period after the launch of the Israeli COVID-19 vaccination campaign. In addition, the study tested whether the observed gains in abnormal findings may be linked to PCS or COVID-19 vaccination.

**Methods:**

Our data set included 572,480 ambulatory medical imaging patients in a national health organization from January 1, 2019, to August 31, 2021. We compared different measures of medical imaging utilization and clinical findings therein before and after the surge of the pandemic to identify significant changes. We also inspected the changes in the rate of abnormal findings during the pandemic after adjusting for changes in medical imaging utilization. Finally, for imaging classes that showed increased rates of abnormal findings, we measured the causal associations between SARS-CoV-2 infection, COVID-19–related hospitalization (indicative of COVID-19 complications), and COVID-19 vaccination and future risk for abnormal findings. To adjust for a multitude of confounding factors, we used causal inference methodologies.

**Results:**

After the initial drop in the utilization of routine medical imaging due to the first COVID-19 wave, the number of these exams has increased but with lower proportions of older patients, patients with comorbidities, women, and vaccine-hesitant patients. Furthermore, we observed significant gains in the rate of abnormal findings, specifically in musculoskeletal magnetic resonance (MR-MSK) and brain computed tomography (CT-brain) exams. These results also persisted after adjusting for the changes in medical imaging utilization. Demonstrated causal associations included the following: SARS-CoV-2 infection increasing the risk for an abnormal finding in a CT-brain exam (odds ratio [OR] 1.4, 95% CI 1.1-1.7) and COVID-19–related hospitalization increasing the risk for abnormal findings in an MR-MSK exam (OR 3.1, 95% CI 1.9-5.3).

**Conclusions:**

COVID-19 impacted the use of ambulatory imaging exams, with greater avoidance among patients at higher risk for COVID-19 complications: older patients, patients with comorbidities, and nonvaccinated patients. Causal analysis results imply that PCS may have contributed to the observed gains in abnormal findings in MR-MSK and CT-brain exams.

## Introduction

COVID-19 had a large impact on the utilization of health care services, with a considerable drop in patient visits during the initial phases of the pandemic [1,2]. The extent of the decrease varied depending on procedure and patient characteristics [3,4]. A key reason for avoidance of medical care was the risk of coronavirus infection. This risk was mitigated by COVID-19 vaccines during the later stages of the pandemic [5]. Despite the demonstrated effectiveness of COVID-19 vaccines in preventing severe disease, large subpopulations have remained wary of the vaccines [6].

COVID-19 had an immense impact on many aspects of daily life, which can influence observed health outcomes. First, changes in the utilization of medical care can affect the number of diagnosed clinical conditions. Indeed, there have been multiple reports on lower diagnosis rates of cancer during the pandemic [7-9]. Second, SARS-CoV-2 infection is associated with a broad spectrum of clinical symptoms manifested in multiple organ systems [10] with potential persistent and prolonged effects known as long COVID or postacute COVID-19 syndrome (PCS) [11]. Finally, many studies reported changes in lifestyle and mental health during the pandemic [12], which may have also affected observed health outcomes.

Therefore, we studied the impact of the COVID-19 pandemic on the utilization of outpatient medical imaging exams and the clinical findings that were observed therein, focusing mainly on magnetic resonance (MR) imaging, computed tomography (CT), and mammography (MG) exams. This study leveraged longitudinal data from a large nationwide cohort of 572,480 ambulatory medical imaging patients aged 18 years and older. These data were extracted from the electronic health records (EHRs) of the second largest health maintenance organization (HMO) in Israel during the period between January 1, 2019, and August 31, 2021. Our data set included information on patient demographics, socioeconomic status, comorbidities, history of health care utilization, COVID-19 test results, and COVID-19 vaccinations. In addition, we augmented the EHR data with public data sets that included daily measures of COVID-19 morbidity, nonpharmaceutical interventions, and changes in the mobility of the population.

**Figure 1:**
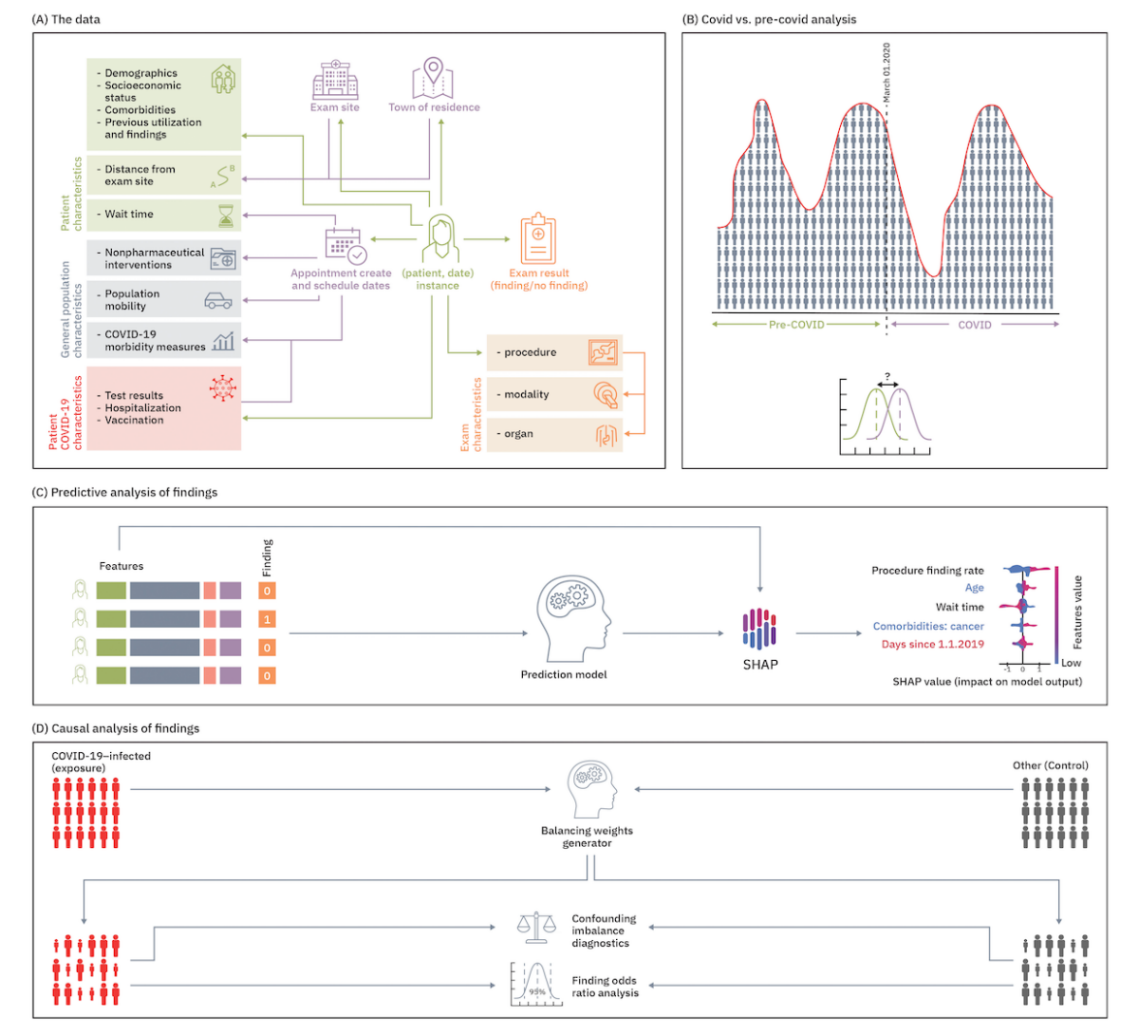
Overview of the data and analyses in the study: (A) data elements per patient with an imaging exam (exam results were given only for mammography, computed tomography, and magnetic resonance exams); (B) statistical analysis of the change in daily utilization and findings measures between the COVID and pre-COVID periods; (C) predictive analysis of exams with findings, with feature importance estimations by the Shapley Additive exPlanations (SHAP) package; (D) causal analysis of the effect of COVID-19 infection, hospitalization, and vaccination on the risk for findings, adjusted for all observed potential confounding factors (eg, those identified as predictive for exam findings).

An overview of the data and various analyses in this study is displayed in Figure 1. First, we analyzed changes in medical imaging utilization during the pandemic across different patient subgroups (Figure 1B). We used predictive analysis to test the link between observed patient characteristics and the risk for abnormal clinical findings (Figure 1C). Finally, we estimated the causal effect of SARS-CoV-2 infection, COVID-19–related hospitalization (indicative of COVID-19 complications), and COVID-19 vaccination on the identified increases in abnormal imaging findings (Figure 1D). 

## Methods

### Data

Our study utilized data from Assuta Medical Centers (ASMC) and Maccabi Healthcare Services (MHS). MHS is the second largest HMO currently active in Israel, representing 25% of the Israeli population. ASMC is a subsidiary of MHS and the largest private network of hospitals in Israel. The data covered all imaging exams of MHS patients that were performed in ASMC from January 1, 2019, to August 31, 2021. The data set had demographic and clinical information, including COVID-19 tests, hospitalizations, and vaccines. Data on patients’ comorbidities were extracted from the registries created and maintained by MHS based on each patient’s central medical record. These comorbidities included cancer, hypertension [13], diabetes [14,15], cardiovascular diseases [16], chronic kidney disease [17], abnormal BMI, and chronic obstructive pulmonary disease. Additionally, the data included Charlson comorbidity index (CCI) [18] scores from the years 2018 and 2020. Finally, the data set also had information on non-imaging health care utilization, such as doctor visits and hospitalizations, in the 90 days before the imaging exam.

For MG, CT, and MR imaging exams, we had access to the results of the exams entered by radiologists. Each MG, CT, and MR result was classified as either “finding” or “no-finding.” MG results included cancer risk scores using the American College of Radiology Breast Imaging Reporting and Data System (BI-RADS) lexicon. We classified an MG result as “finding” if the BI-RADS score was ≥4, which indicates an abnormal finding that is suspicious for cancer; BI-RADS 1 to 3 were classified as “no-finding.” CT and MR exams were classified as “finding” if the radiologist entered one of the following options: “abnormal finding,” “urgent finding-24 hours,” “life-threatening finding.” Alternatively, CT and MR exams were labeled “no finding” if the radiologist entered “no finding” or “standard reporting that is not special.” The information on CT and MR exams also specified the organ system that was examined.

### Study Design and Population

Our study population included all MHS patients who had an outpatient imaging exam in ASMC during the period between January 1, 2019, and August 31, 2021, and were aged 18 years or older at the time of the exam. For complete details of the cohort selection procedure, see Multimedia Appendix 1.

**Figure 2:**
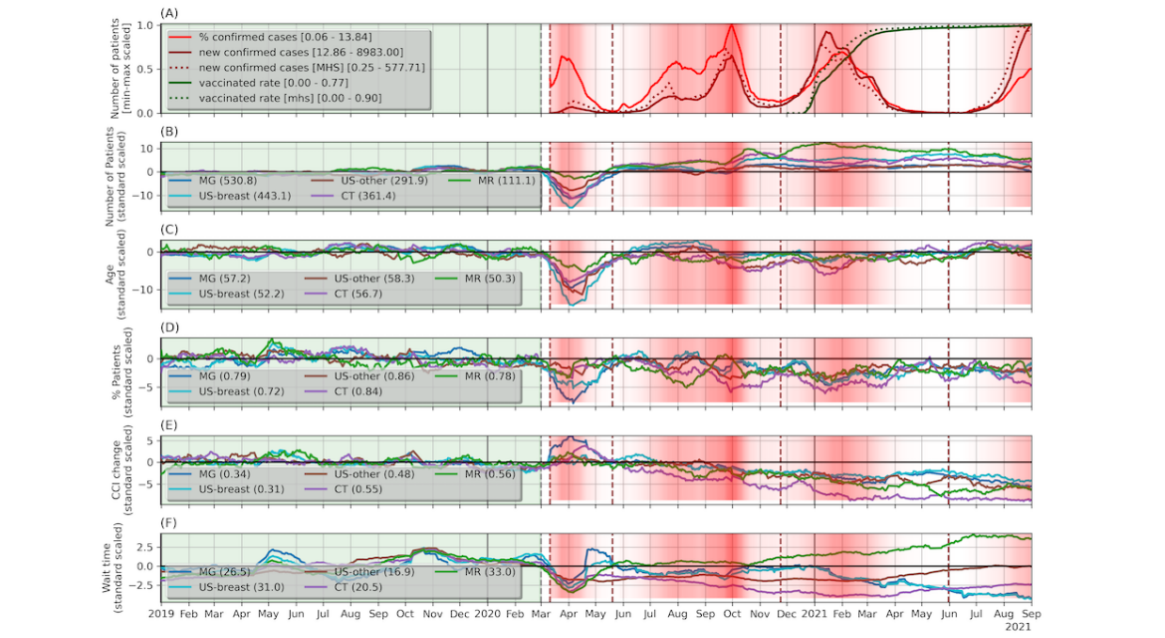
Timeline of scaled medical imaging measures, with green and red backgrounds denoting pre-COVID-19 and COVID-19 periods, respectively; the COVID-19 wave boundaries are indicated by red dashed lines, and the intensity of the red color indicates the level of COVID-19 morbidity. Square brackets in the legend specify the [min, max] scaling constants; round brackets specify the subtracted mean in standard scaling. All measures were smoothed by taking the average of a centered 28-days window. (A) COVID-19 spread measures and vaccination rate; daily utilization measures computed with respect to the set of unique patients with exams on each date and normalized with standard scaling using the mean and SD values computed during the pre-covid period, including (B) daily number of patients, (C) daily average age, (D) percentage of patients per day with any comorbidities from the list (cancer, hypertension, diabetes, cardiovascular diseases, chronic kidney disease, abnormal BMI, and chronic obstructive pulmonary disease), (E) daily average of the change in Charlson comorbidity index (CCI) scores during 2018 and 2020, and (G) average wait time (if a patient had multiple exams on a date, the maximal wait time was used). CT: computed tomography; MG: mammography; MR: magnetic resonance; US-breast: breast ultrasound; US-other: other nonbreast ultrasound.

We referred to the time prior to March 1, 2020, as the “pre-covid period” and the time that started at this date as the “covid-period.” The partition of the covid-period into the time periods of the 4 COVID-19 waves was based on the daily rate of COVID-19 confirmed cases (see Figure 2). Here are the computed endpoints for each wave: wave1, March 1, 2020, to May 19, 2020; wave2, May 20, 2020, to November 23, 2020; wave3, December 24, 2020, to May 30, 2021; wave4, May 31, 2021, to August 31, 2021 (end of the data).

The impact of COVID-19 on medical imaging utilization and findings was evaluated by comparing different daily measures between the covid-period and pre-covid period. The daily measures considered only unique patients per day, aggregating multiple exams of the same patient. These included the number of patients, number of patients with findings, finding rate (obtained by the ratio of the 2 previous measures), average age, percentage of patients with comorbidities, average patient CCI scores, and average wait time (using the maximal wait time per patient).

We excluded from the analysis of daily measures weekends and additional dates showing extreme drops in the use of medical imaging services due to various reasons (see Multimedia Appendix 1 for more details). The change between the covid-period and pre-covid period in the rate of clinical findings was additionally assessed by applying a proportion test on the set of (patient, date) visits.

### Feature Extraction

We used our feature extraction tool [19] to extract features for each (patient, date) visit. These included patient characteristics (eg, age, gender, comorbidities), exam-related features (eg, procedure and organ system indicators, wait time), and time-dependent features (eg, COVID-19 daily measures). For the complete set of features that was extracted, see Multimedia Appendix 1. These features were used to compute daily measures (eg, average age), subgroup analysis (eg, CT-chest exams), predictors in risk models for clinical findings (see the next section), and potential confounding factors when estimating causal associations.

### Risk Models for Clinical Findings

We trained prediction models to estimate the risk of a clinical finding for a (patient, date) visit using the XGBoost [20] package. For additional details on the development of these models and the set of features they used, see Multimedia Appendix 1. To identify important features in these models, we used TreeExplainer from the Shapley Additive exPlanations (SHAP) [21]. This analysis was used to provide insight on confounding factors for the estimation of causal effects on clinical findings.

### Statistical Analysis

In the comparison of daily measures between the covid-period and pre-covid period, as well as in the comparison of vaccinated and vaccine-hesitant groups, we assessed the significance of the difference in the distribution of the compared variable (eg, the finding rate) with a 2-tailed *t* test with unequal variance.

We adjusted for confounding factors by applying one of these methods to generate balancing weights: inverse probability weighting (IPW) [22,23] and adversarial balancing (AdvBal) [24]. Both methods used logistic regression analysis, with preprocessing of removing constant features and applying standard scaling. AdvBal was run with 20 iterations (the default). All the statistical tests that evaluated the effect of COVID-19 were performed 3 times: (1) unadjusted for confounding factors, (2) adjusted with IPW, and (3) adjusted with AdvBal.

We measured the bias between 2, possibly weighted, groups by computing the standardized mean difference (SMD):




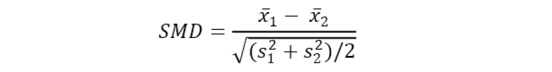




where 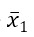 and 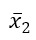 are the feature means in the 2 groups and *s*_1_^2^ and *s*_2_^2^ are the corresponding sample variances. When |SMD|<0.1, the bias is commonly regarded as negligible [22], while others [25] also consider an |SMD|<0.25 as a reasonable cutoff. The estimation of the causal effects with IPW and AdvBal, as well as the balancing diagnostics of the weights, were done using the IBM causallib package [26].

In the evaluation of the impact of COVID-19 on medical imaging findings, we accounted for changes in the utilization during the pandemic by applying balancing weights for (patient, date) visits in the covid-period only, keeping the weights in the pre-covid period fixed to 1. We generated these weights with IPW in the same manner used for estimating the “average treatment effect in the treated” [22]. For the AdvBal method, this was done by setting the target population to the subset of visits from the pre-covid period. Balancing weights were generated for the covid-period in each of the 4 waves independently, to allow for separate analysis of each of the waves.

We measured the effect of different COVID-19–related exposures (eg, SARS-CoV-2 infection) on the risk for clinical findings with the odds ratio (OR), which was calculated using a logistic regression model. To adjust for confounding factors, we applied balancing weights as the sample weights. The balancing weights were computed using IPW and AdvBal methods.

We used the procedure by Benjamini and Hochberg [27] on all the tests reported in this study, to account for multiple testing using a threshold of 0.05 on the false discovery rate (FDR). *P* values that were found to be significant at an FDR of 0.05 are marked with “*” in Tables S1-S6 in Multimedia Appendix 2, which contain the results of all the statistical tests in this study. The statistical tests were implemented with the SciPy [28] and statsmodels [29] packages.

### Ethical Considerations

This retrospective study was reviewed and approved by the institutional review board at ASMC (0033-20-ASMC), which waived the requirement for patient consent. All the data analyzed in this study were anonymous, with no personal identifiers collected from participants. No compensation was provided to the human participants who participated in the study.

## Results

### Study Cohort

Our study cohort included 572,480 patients who had a total of 1,150,736 days of visits at ASMC. Each visit corresponds to a pair of (patient, date) and contains information about the patient and the one or more medical imaging exams the patient underwent on that date. The most common modalities in our data set included MG, breast ultrasound (US-breast), other nonbreast ultrasound (US-other), CT, and MR imaging. These 5 classes together constitute the bulk of outpatient imaging exams in ASMC, covering 98.9% (566,230/572,480) of the patients and 96% (1,104,764/1,150,736) of the (patient, date) visits in the data set. The analysis of clinical findings was performed for MG, CT, and MR exams, which covered 84.3% (482,404/572,480) of the patients in the data.

The characteristics of the entire study cohort and the 5 classes are presented in Table 1. As shown, the highest (8803/99,396, 8.9%) proportion of patients with findings was observed with MR, and the lowest (9635/262,830, 3.7%) proportion was observed with MG. Most (279,022/370,455, 75.3%) of the MG exams were routine breast cancer screening procedures, with a finding rate of 2% (5577/278,088); the remaining (nonscreening) MG exams had a finding rate of 5.9% (4896/82,292). Patients undergoing US-other and CT exams showed a higher degree of comorbidities and death rate. Higher rates of SARS-CoV-2 infections and COVID-19–related hospitalizations were observed with CT and MR patients, for which these COVID-19–related events could have occurred prior to or after the exams. An illustration of daily COVID-19 morbidity measures and vaccine rate in our study cohort, as well as in the global population in Israel, are given in Figure 2A. As shown, there was a high correlation between the local measures in our study cohort and global measures.

**Table 1:** Characteristics of the study cohort (n=572,480), computed at the end of the study for the set of unique patients.

Characteristics		All	Mammography (n=264,058)	Breast ultrasound (US; n=215,875)	Other nonbreast US (n=141,366)	Computed tomography (n=239,255)	Magnetic resonance (n=100,017)
**Exams**							
	Number^a^, mean (SD)	2.0 (1.5)	1.4 (0.6)	1.5 (0.8)	1.5 (0.9)	1.3 (0.7)	1.3 (0.7)
	Had results^b^, n (%)	482,404 (84.3)	262,830 (99.5)	0 (0)	0 (0)	12,506 (99.9)	99,396 (99.4)
	Had findings^b^, n (%)	28,578 (5.9)	9635 (3.7)	N/A^c^	N/A	238,913 (5.2)	8803 (8.9)
	Wait time (days)^d^, mean (SD)	22 (22)	24 (25)	27 (26)	15 (14)	17 (16)	38 (37)
Gender (female), n (%)		387,986 (67.8)	261,892 (99.2)	212,418 (98.4)	77,805 (55)	125,473 (52.4)	51,977 (52)
Age at first exam (years), mean (SD)		53 (15)	56 (10)	51 (13)	56 (17)	55 (16)	50 (16)
**Marital status, n (%)**							
	Married	295,586 (51.6)	162,505 (61.5)	115,790 (53.6)	75,767 (53.6)	123,548 (51.6)	47,752 (47.7)
	Single	214,912 (37.5)	68,256 (25.8)	77,188 (35.8)	49,670 (35.1)	88,039 (36.8)	43,485 (43.5)
	Divorced	29,403 (5.1)	18,568 (7)	11,745 (5.4)	7693 (5.4)	13,018 (5.4)	4064 (4.1)
	Widowed	6948 (1.2)	3616 (1.4)	1930 (0.9)	2260 (1.6)	3681 (1.5)	723 (0.7)
Socioeconomic score (SES), mean (SD)		6.7 (1.8)	6.8 (1.8)	7.0 (1.8)	6.8 (1.9)	6.5 (1.8)	7.0 (1.8)
**Comorbidities, n (%)**							
	BMI^e^	402,646 (70.3)	187,794 (71.1)	138,115 (64)	107,215 (75.8)	178,785 (74.7)	69,752 (69.7)
	Hypertension	167,298 (29.2)	75,374 (28.5)	42,726 (19.8)	54,692 (38.7)	84,151 (35.2)	25,369 (25.4)
	Diabetes	72,414 (12.6)	29,513 (11.2)	15,040 (7)	24,318 (17.2)	37,862 (15.8)	10,855 (10.9)
	CKD^f^	93,480 (16.3)	38,795 (14.7)	24,194 (11.2)	33,110 (23.4)	47,920 (20)	14,603 (14.6)
	Cardio^g^	68,916 (12)	20,158 (7.6)	13,280 (6.2)	26,278 (18.6)	39,253 (16.4)	12,001 (12)
	Cancer	72,911 (12.7)	35,143 (13.3)	28,183 (13.1)	20,497 (14.5)	32,906 (13.8)	15,560 (15.6)
	COPD^h^	18,398 (3.2)	6892 (2.6)	3845 (1.8)	6034 (4.3)	12,260 (5.1)	2819 (2.8)
	Any of the above	439,627 (76.8)	205,702 (77.9)	152,261 (70.5)	116,689 (82.5)	193,926 (81.1)	76,404 (76.4)
**Charlson comorbidity index (CCI), mean (SD)**							
	2020	1.4 (1.9)	1.0 (1.5)	0.9 (1.5)	1.6 (2)	1.5 (2)	1.2 (1.8)
	2018	0.9 (1.5)	0.7 (1.3)	0.6 (1.2)	1.1 (1.7)	1.0 (1.7)	0.8 (1.3)
Died during the study, n (%)		6517 (1.1)	937 (0.4)	630 (0.3)	1835 (1.3)	3699 (1.5)	1021 (1)
**COVID-19 status, n (%)**							
	Vaccinated	520,819 (91)	246,596 (93.4)	200,766 (93)	129,013 (91.3)	216,243 (90.4)	91,399 (91.4)
	Infected	52,155 (9.1)	20,674 (7.8)	18,286 (8.5)	12,328 (8.7)	22,683 (9.5)	9248 (9.2)
	Hospitalized	3093 (0.5)	1026 (0.4)	856 (0.4)	1043 (0.7)	1888 (0.8)	798 (0.8)

^a^Number of exams on distinct dates.

^b^In at least one exam.

^c^N/A: not available because the results were not available for these imaging modalities.

^d^For patients with multiple exams, we used the average wait time.

^e^Abnormal BMI.

^f^CKD: Chronic kidney disease.

^g^Cardio: Cardiovascular diseases.

^h^COPD: Chronic obstructive pulmonary disease.

### Changes in Medical Imaging Utilization During COVID-19

**Figure 3:**
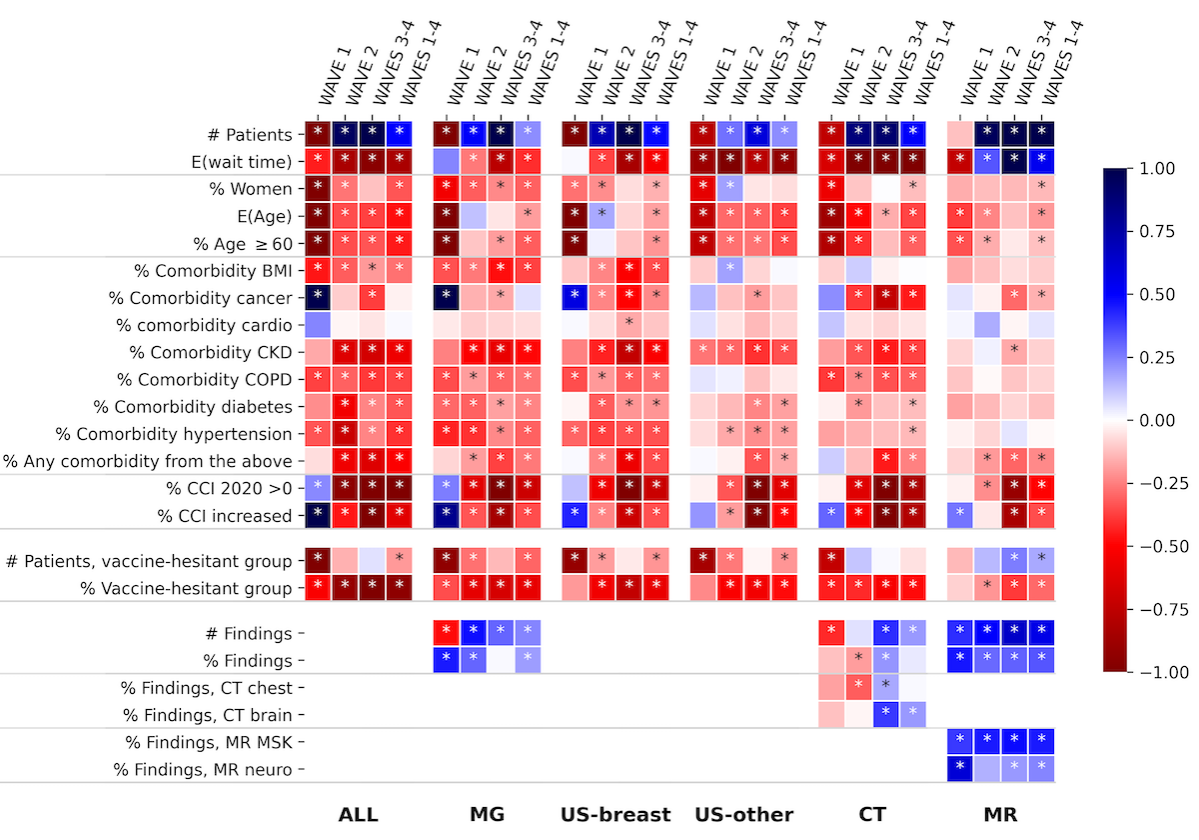
Changes in daily measures of medical imaging utilization and abnormal findings, with the colors indicating the change in the daily measure, calculated as the standard mean difference (SMD) from the pre-covid period, and significant changes (*P*<.05) indicated with “*” in the boxes. Tables S1, S3, and S5 in Multimedia Appendix 2 list the underlying values, and similar results were obtained after correcting for the observed biases in medical imaging utilization during the covid-period (see Figure S3 in Multimedia Appendix 1). cardio: cardiovascular diseases; CCI: Charlson comorbidity index; CKD: chronic kidney disease; COPD: chronic obstructive pulmonary disease; CT: computed tomography; MG: mammography; MR: magnetic resonance; MSK: musculoskeletal; US-breast: breast ultrasound; US-other: other nonbreast ultrasound.

We analyzed the changes in the use of medical imaging services, as these have the potential to affect the number and rate of the detected clinical findings. Figures 2B-2F illustrate the changes in various daily utilization measures during the covid-period, with respect to the pre-covid period. We applied statistical tests to assess the significance of the changes in these daily measures. The results of these tests are presented in Table S1 in Multimedia Appendix 2 and visualized at the top section of Figure 3. In the following paragraphs, we provide a summary of these results.

The first COVID-19 wave was clearly distinct, showing major decreases in the number of exams performed, average age, and average number of comorbidities (Figures 2B-2D). Despite the decrease in the utilization during the first wave, when examining the entire covid-period, we saw an overall significant increase in the daily number of exams, with an SMD of 0.5 (*P*<.001). An inspection of the composition of the population during the entire covid-period indicated that, overall, patients were younger (SMD=–0.47, *P*<.001) and with fewer comorbidities (eg, SMD of having any comorbidity from the defined list=–0.57, *P*<.001); in addition, the proportion of women was lower (SMD=–0.33, *P*<.001). These trends started in the second wave and persisted during the time period that followed (waves 3-4), with the exception of the percentage of women, which almost returned to its prepandemic levels (SMD=–0.12, *P*=.06). The decrease in the proportion of patients with comorbidities remained statistically significant also after adjusting for age and gender biases.

MG patients that underwent medical imaging exams during the first wave showed a larger increase in their CCI scores, suggesting that these exams might be related to the deterioration in their clinical state (Figure 2E). Another noteworthy observation is that, during the covid-period, the average wait time for medical imaging exams dropped substantially despite the increase in the number of exams conducted (Figure 2F). The only exception was MR wait times, which significantly increased, mainly after the second wave (Figure 2F).

### Vaccine-Hesitant Patients

The Israeli COVID-19 vaccination campaign was launched on December 20, 2020, and 3 months later, 81% of the population aged 16 years and older had received at least one vaccination [30]. We obtained the patients’ vaccination data as of October 10, 2021, more than 9 months after the launch of the vaccine campaign in Israel. By that time, 91% (520,819/572,480) of the patients in our data set had received the first vaccine, and 83% (432,125/520,819) of those had already received a third dose (“booster”). The vast majority (497,578/520,819, 95.5%) of the vaccinated patients in this study had their first dose by the end of March 2021. Here, we analyzed the group of patients who were not vaccinated by the end of our study (“vaccine-hesitant population”) by comparing with the group of patients who did receive the vaccine (“vaccinated population”). We inspected the differences in the characteristics of the 2 groups, as well as their history of medical imaging use. This analysis included only patients who were alive at the time of the comparison (ie, the end of the study).

Of the 572,480 patients, the vaccinated and vaccine-hesitant groups included 519,105 (90.7%) and 46,858 (8.2%) patients, respectively. In our data, 21,410 (4.1%) of the 520,819 vaccinated patients received the vaccine after testing positive for COVID-19, in agreement with the recommendation to vaccinate patients 5 months after SARS-CoV-2 infection. The vaccine-hesitant group was, on average, of a lower socioeconomic status and younger age; fewer of this group was married, they had fewer comorbidities, and they lived further from their exam facility (Table S2 in Multimedia Appendix 2). Additionally, the vaccine-hesitant group had a larger number of confirmed cases (10,778/46,858, 23% vs 40,849/519,105, 7.9%) and COVID-19–related hospitalizations (771/46,858, 1.6% vs 2234/519,105, 0.4%) than the vaccinated group.

Analysis results of the daily utilization of medical imaging among the vaccine-hesitant group appear in Figure 3, Table S3 in Multimedia Appendix 2, and Figure S2 in Multimedia Appendix 1. The daily proportion of the vaccine-hesitant group significantly dropped during the covid-period for each imaging modality. The largest decrease was observed with MG (SMD=–0.62, *P*<.001), and the lowest was observed with MR (SMD=–0.22, *P*<.001). Additionally, the daily number of patients was significantly lower during the covid-period, with the exception of patients undergoing CT and MR modalities.

### Changes in Clinical Findings During COVID-19

**Figure 4:**
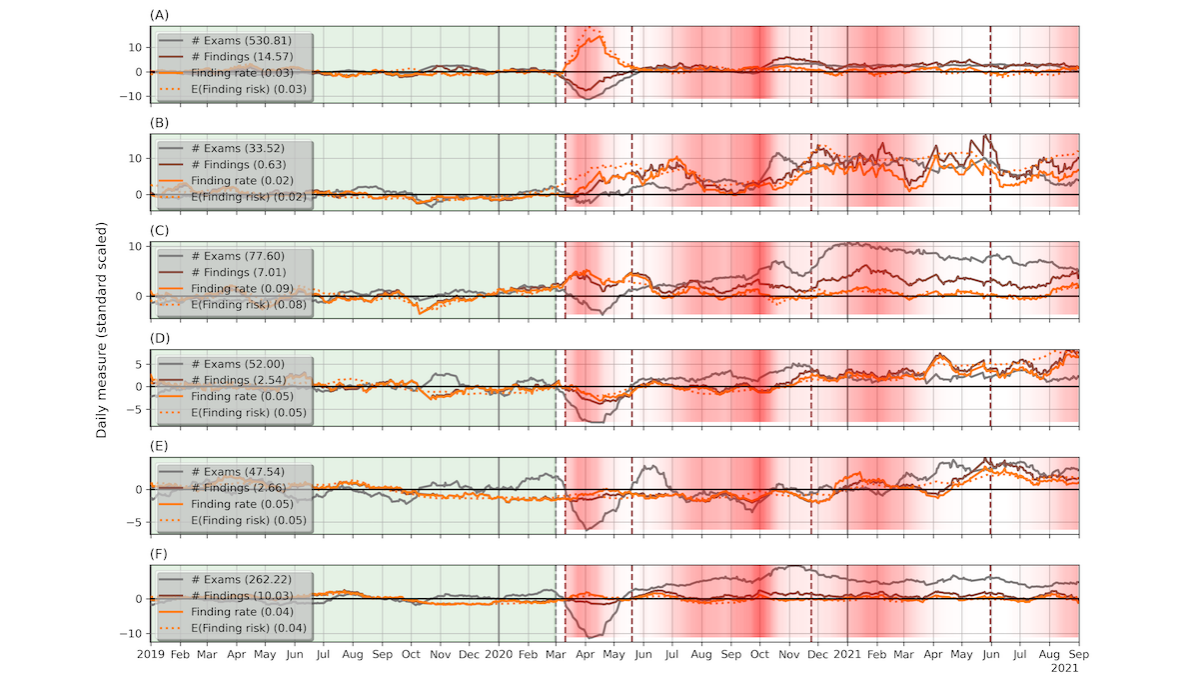
Daily measures of exams, findings, finding rate, and finding risk, standard scaled with the mean and SD during the pre-covid period, smoothed by taking the average of a centered 28-day window, for (A) mammography, (B) musculoskeletal magnetic resonance (MR-MSK), (C) MR without MSK, (D) computed tomography (CT)-brain, (E) CT-chest, and (d) CT without brain or chest. The subtracted mean values are shown in the legends.

Figure 4 illustrates the daily average finding risk for MG, CT, and MR patients estimated with our prediction models, along with the daily rate of findings, revealing a remarkable agreement between the 2 measures. The complete results of our investigation of the impact of COVID-19 on the number and rate of detected clinical findings in MG, CT, and MR exams appear in Table S5 in Multimedia Appendix 2 and are visualized in Figure 3. In the following paragraphs, we summarize the results of the unadjusted tests. By and large, these results remained also after correcting for the observed biases in medical imaging utilization during the covid-period.

The daily number of patients with findings on MG, CT, and MR significantly increased during the covid-period, as may be expected by the corresponding gains in the daily number of exams. We therefore analyzed the impact of COVID-19 on the finding rate, which is the daily number of patients with findings normalized by the daily number of patients. We analyzed changes over the entire covid-period as well as within its 3 subperiods: wave 1, wave 2, and waves 3-4. We made this partition because the first wave exhibited distinct changes in the utilization and COVID-19 vaccines became available at the beginning of the third wave.

When considering the entire covid-period, we observed a significant rise in the rate of findings on MR exams (4228/48,931, 8.6% during the covid-period vs 2120/30,559, 6.9% during the pre-covid period; *P*<.001) and smaller, yet statistically significant, increases in MG exams (5845/195,724, 3% during the covid-period vs 4039/144,019, 2.8% during the pre-covid period; *P*<.001) and CT exams (6241/142,027, 4.4% during the covid-period vs 4227/100,550 4.2% during the pre-covid period; *P*=.01). We then tested whether the increases in MR and CT findings could be attributed to specific organ systems. We initially analyzed MR, as it showed the most significant increase in the finding rate. We partitioned MR exams into 4 subclasses: neuro (brain and nerve system), musculoskeletal (bones, soft tissues, and joints), breast, and body (abdomen, heart, and other organs not included in previous classes). For CT, we examined chest and brain exams, as these were shown to have manifestations of COVID-19 disease. The results for each of these exam classes are shown in Figure 4 and Table S5 in Multimedia Appendix 2. In the following paragraphs, we provide a summary of these results.

Overall, during the entire covid-period, MR of the musculoskeletal system (MR-MSK) showed an outstanding increase in the finding rate, from 1.9% (176/9288) to 5.6% (864/15,485; *P*<.001), and an elevated finding rate was observed in each of the tested covid-period subperiods. Prominent gains were also observed in the finding rate on CT-brain exams (1224/19,730, 6.2% vs 715/14,504, 4.9%; *P*<.001) and MR-neuro exams (1594/23,473, 6.8% vs 811/14,478, 5.6%; *P*<.001). On the other hand, we observed no significant change in the finding rate on CT-chest exams (1002/17,589, 5.7% vs 741/13,251, 5.6%; *P*=.30). Additional investigation revealed that MR-MSK patients were relatively young (mean 46, SD 15 years) and with fewer comorbidities (eg, mean CCI 2020 0.7, SD 1.2), while CT-brain patients were mostly women (15,542/25,724, 60.4%) and of relatively older age (mean 57, SD 18 years). See Multimedia Appendix 1 for additional analysis results of these patient groups.

### Causal Associations Between Clinical Findings and SARS-CoV-2 Infection, COVID-19–Related Hospitalization, and COVID-19 Vaccination

To test whether the observed growth in MR, CT, and MG abnormal findings could be linked to SARS-CoV-2 infection, hospitalization (indicating COVID-19 complications), or vaccination cases, we tested the causal effect of these exposures on the finding rate. SARS-CoV-2 infection was defined as having a positive COVID-19 test result. We limited the time period of the visits in these tests to waves 3-4, since before that, vaccines were not available and the number of visits whose patients were previously infected or hospitalized with COVID-19 was relatively small. We tested additional subclasses of MR and CT that showed higher finding rates during waves 3-4: MR-MSK, MR-neuro, CT-brain, and CT-chest (see Table S5 in Multimedia Appendix 2). In the analysis of COVID-19–related hospitalizations, we excluded the variable “number of hospitalizations in the last 90 days” from the set of potential confounders. For the analysis of COVID-19 vaccinations, we added the exposure variables of previous SARS-CoV-2 infection and COVID-19–related hospitalization as confounders.

**Figure 5:**
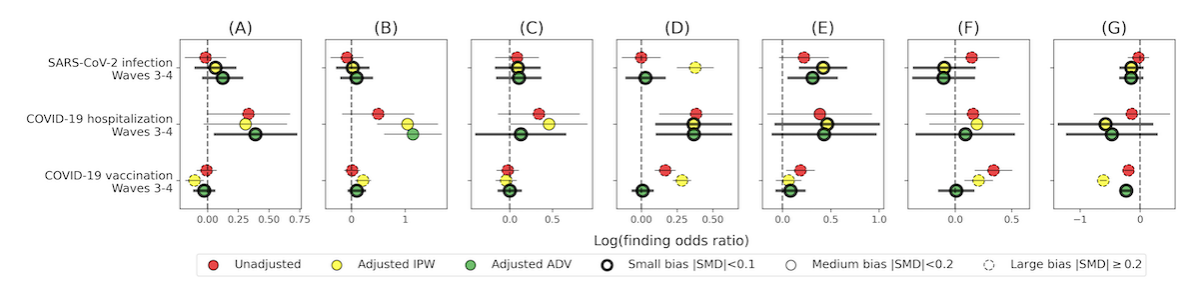
Estimated causal effects of SARS-CoV-2 infection, COVID-19–related hospitalization, and COVID-19 vaccination on the finding rate in different imaging classes. ADV: adversarial balancing; IPW: inverse probability weighting; SMD: standardized mean difference.

The estimated effects, as well as balancing statistics, appear in Figure 5 and Table S6 in Multimedia Appendix 2. In all our tests, AdvBal was superior to the IPW method in minimizing the absolute max bias, which was measured using the SMD. Nevertheless, when both IPW and AdvBal managed to eliminate the bias, their estimations were in agreement. In the following paragraphs, we report only the estimations from the AdvBal method.

Our results indicated that SARS-CoV-2 infection had a significant effect on increasing the finding rate only in CT-brain exams (OR 1.36, 95% CI 1.07-1.75). COVID-19–related hospitalization demonstrated a stronger effect on increasing the finding rate in MR, MR-MSK, and CT exams, with MR-MSK showing the strongest effect (OR 3.14, 95% CI 1.85-5.33). In CT-brain exams, the estimated effect of COVID-19–related hospitalization was higher than for SARS-CoV-2 infection but with larger variance (OR 1.54, 95% CI 0.91-2.6). The estimated causal effects of COVID-19 vaccination were significant only for MG exams, indicating a reduced rate of mammogram findings for vaccinated patients (OR 0.79, 95% CI 0.74-0.85).

## Discussion

In this study, we leveraged a large nationwide cohort of patients who underwent medical imaging examinations during the period between January 1, 2019, and August 31, 2021, to investigate the impact of COVID-19 on medical imaging utilization and the detection of abnormal findings therein. The correlation between our cohorts’ statistics for COVID-19 morbidity and vaccination rates, with the statistics computed at the country level, further supports the assumption that our study cohort represents the global population of health care consumers in Israel.

### Principal Findings

We observed an overall increase in the use of medical imaging exams during the covid-period but with a lower proportion of older patients, patients with comorbidities, women, and vaccine-hesitant individuals, compared with the pre-covid period. Furthermore, our results indicate a significant increase in the rate of abnormal imaging findings among the general population, specifically in MR-MSK and CT-brain exams. After adjusting for a variety of potentially confounding factors, our results demonstrated 2 possible causal associations: (1) SARS-CoV-2 infection leading to increased risk for abnormal findings in CT brain exams and (2) COVID-19–related hospitalizations leading to increased risk of abnormal findings in MR-MSK exams. Additionally, COVID-19 vaccination was associated with a lower rate of abnormal findings in MG exams, also after accounting for confounding variables.

The increase in the number of exams, along with the overall reduction in patients’ wait times, suggests a more efficient use of medical imaging resources during the pandemic. This is aligned with worldwide reports on the need for health care professionals and managers to act quickly to mitigate the repercussions of COVID-19 on the provision of health care services [31-33]. The strong association of shorter wait times with higher risk for clinical findings indicates adequate prioritization of exams by expected level of urgency. We conjecture that the improvement in medical imaging utilization enabled the treatment of a larger number of urgent cases, which otherwise would have been treated at inpatient hospitals.

The subpopulations who showed a lower degree of medical imaging utilization during the pandemic included women, older patients, patients with comorbidities, and vaccine-hesitant individuals. Age, comorbidities, and nonvaccination are risk factors for COVID-19 complications, and therefore, the aforementioned subpopulations may have refrained from attending public places, including medical facilities. Health care avoidance during COVID-19 was previously observed for women [3,34]. The vaccine-hesitant population had distinct demographic and socioeconomic characteristics compared with the vaccinated population. These included younger age, lower socioeconomic status, and further distance from a medical facility, which were in agreement with previous studies on vaccine hesitancy [30,35,36]. We accounted for such differences in patients’ characteristics when we evaluated the impact of the COVID-19 pandemic and its vaccines on the rates of abnormal imaging findings.

Vaccines were shown to be associated with lower probability for abnormal findings in MG exams, after adjusting for all observed confounding factors. We conjecture that nonvaccinated patients showed higher rates of MG findings due to their greater tendency to avoid MG exams when the estimated risk for abnormal findings is low. The phenomenon of decreased MG utilization among people with lower clinical risk was observed in our data for the general population during the first wave. It is consistent with previous studies of health care utilization during the initial phase of the pandemic, which showed a greater decrease in utilization among patients with less severe illnesses [1,37].

MR-MSK exams are primarily used to evaluate the bones, soft tissues, and joints for injuries, tumors, and degenerative diseases. In our data set, MR-MSK patients were, on average, 46 (SD 15) years old, with relatively lower rates of morbidity, and of higher socioeconomic status. Hence, we hypothesize that the observed increase in abnormal MR-MSK findings may be associated with changes in lifestyle and physical activity during the pandemic, as has been recently reported in multiple studies [38-41]. The significant association between abnormal MR-MSK findings and COVID-19–related hospitalizations may suggest that some of the increase in abnormal findings during the pandemic may also be related to MSK manifestations of severe cases of COVID-19.

The increase we observed in the rate of abnormal CT-brain findings during COVID-19, together with the significant estimated causal effect of COVID-19 infection on increasing the risk for abnormal CT-brain findings, suggests that some of these findings may be related to neurological complications of COVID-19. At its outset, COVID-19 was mainly associated with acute respiratory syndromes, which had clinical manifestations on CT-chest scans [42,43]. Since patients suspected of having COVID-19 were not allowed to enter ASMC exam facilities, we believe that the decrease in CT chest findings during waves 1 and 2 was associated with this policy. Despite the observed increase in CT findings during waves 3 and 4, our causal analysis of CT-chest exams did not find any significant association between previous SARS-CoV-2 infection or COVID-19–related hospitalization and the risk for CT-chest findings.

### Limitations

Our study had several limitations. First, the information we had on clinical findings is mostly indicative: Apart from the involved organ system, we had no data on the type and nature of the detected abnormalities. In addition, we did not have information on the reasons for the referral to imaging exams. The availability of data on the type of clinical finding or the reasons for referral could shed light on the true causes for the observed increase in the rates of abnormal imaging findings. Second, our data set was derived from EHRs and hence may be noisy and incomplete. Third, our causal analysis of SARS-CoV-2 infection, COVID-19–related hospitalization, and COVID-19 vaccination is likely to be missing confounders, such as additional factors related to the clinical and socioeconomic state of the patients. A common limitation in all studies of causal effects is the inability to measure the accuracy of the estimated effects, due to the lack of ground truth. Finally, our data set was derived from a single HMO in Israel; patient characteristics and medical exam policies may differ for other HMOs and geographies.

### Comparison With Prior Work

A recent study on PCS patients in our health care organization [44] revealed significant gains in health care utilization among PCS patients, with doubled direct medical costs for patients with long COVID symptoms, compared with prior to the SARS-CoV-2 infection. This suggests that the gains we reported in medical imaging exams may be also due to post-COVID-19 patients and those exhibiting long COVID symptoms.

SARS-CoV-2 infections were associated with neurological complications, including anosmia (loss of smell), ageusia (loss of taste), headache, and stroke [45,46]. Brain imaging results of neuro-COVID-19 patients were shown to manifest a spectrum of clinical findings [47-49]. Alterations in brain structure were also demonstrated in mild cases of COVID-19 a few months after the infection [50]. There is ongoing research on the brain imaging manifestations of post-COVID-19 neurological syndrome (PCNS), with multiple reports on alterations in CT scans of the brain in PCNS patients [51].

COVID-19 is also associated with an array of MSK disorders, including fatigue, muscle pain, back pain, muscle weakness, and joint stiffness [52,53]**.** MR imaging was used to diagnose various COVID-19–related MSK findings, most commonly for patients with prolonged hospitalizations [54,55]. MSK manifestations of COVID-19 may persist or occur months after the initial infection, known as post-COVID syndrome [56].

### Conclusions

We presented a large-scale retrospective cohort study that contributes to the rich body of work on health services utilization during COVID-19. Our results indicate that health care avoidance exists also in the era of COVID-19 vaccinations among patients at risk for COVID-19 complications: elderly patients, patients with comorbidities, and the nonvaccinated. Our analysis of the causal effects of COVID-19 yielded new evidence for the linkage between incidence of SARS-CoV-2 infection and COVID-19–related hospitalization and increased risk for abnormal findings in future MSK and brain imaging exams. To the best of our knowledge, this is the first study that demonstrates the association of a post-COVID condition with increased risk of MSK and brain findings on a nationwide scale. Additional studies are needed to explore whether the trends and associations we observed persisted in the later stages of the pandemic and to further investigate the correlation of the post-COVID condition with increased rates of abnormal imaging findings.

## Appendix

Multimedia Appendix 1 Supplementary file with additional information on methods as well as additional results and figures.

Multimedia Appendix 2 Supplementary tables S1-S6, which contain all the statistical tests performed in this study and an explanation for each table on the readme tab.

## Abbreviations

AdvBal: adversarial balancing
ASMC: Assuta Medical Centers
BI-RADS: Breast Imaging Reporting and Data System
CCI: Charlson comorbidity index
CT: computed tomography
EHR: electronic health record
FDR: false discovery rate
HMO: health maintenance organization
IPW: inverse probability weighting
MG: mammography
MHS: Maccabi Healthcare Services
MR: magnetic resonance
MR-MSK: MR of the musculoskeletal system
OR: odds ratio
PCNS: post-COVID-19 neurological syndrome
PCS: postacute COVID-19 syndrome
SHAP: Shapley Additive exPlanations
SMD: standardized mean difference
US-breast: breast ultrasound
US-other: other nonbreast ultrasound

## Data Availability

Patients’ data from Assuta Medical Centers and Maccabi Health Services are not publicly available, due to Israel Ministry of Health regulations. These data can be accessed only by request to Assuta Medical Centers and Maccabi Health Services. We obtained socioeconomic measures and population sizes for localities in Israel from the Central Bureau of Statistics reports [57]. Time-based statistics of coronavirus morbidity in Israel, at the country and locality levels, were downloaded from the COVID-19 Data Repository of the Israeli Ministry of Health [58]. We downloaded Israel Transverse Mercator coordinates for localities in Israel from the public Israeli Government Databases [59] and used these data to compute pairwise distances between the patients’ towns and exam sites. We obtained data on nonpharmaceutical interventions in Israel, including closures, from the IBM Worldwide Non-pharmaceutical Interventions Tracker for COVID-19 [60]. Finally, we acquired community mobility data in Israel from Google Community Mobility Reports [61].

The causal analysis code, including the inverse probability weighting and adversarial balancing methods, is freely available at [62].
