# Supplementary material for: Impact of the COVID-19 Pandemic on Clinical Findings in Medical Imaging Exams in a Nationwide Israeli Health Organization: Observational Study
Source: JMIR Form Res. 2023 May 2;7:e42930. doi: 10.2196/42930 (PMC10156149; doi:10.2196/42930)
Supplement: Multimedia Appendix 1 [file formative_v7i1e42930_app1.docx]

## Methods: additional information

### Implementation of cohort selection

#### Excluded procedures

Since only the birth year was available for patients, the age at exam time was computed by setting each patient’s birth date to be July 1. We ignored imaging procedures that do not involve an examination by a radiologist, such as imaging guided biopsies. We also ignored encounters of research procedures, or those in which the patient need not be physically present, such as tumor boards and consultations. Finally, we excluded MR-prostate exams, as these were provided as a service for MHS patients from the beginning of the year 2020, independent of the surge of COVID-19 pandemic.

#### Dates excluded from the analysis of daily measures

We excluded from the analysis of daily measures the following days: weekends (Friday-Saturday, as Sunday-Thursday are the working days in Israel), holidays, legislative election days (which are sabbaticals days in Israel), and several days during 2019 and 2021 in which there were missile attacks, causing major drops in the overall imaging utilization.

### Extracted features

The set of features for a (patient, date) visit included the following information: age, gender, socioeconomic status (SES) scores, marital status, distance from the exam facility, procedures, organ systems, pre-exam urgency, wait time (i.e., the time between creation of the encounter record and the exam date), weekend and holiday indicators, comorbidities, CCI scores, 90-days hospitalization history, 90-days findings history, history of COVID-19 test results, history of COVID-19 vaccinations, COVID-19 daily measures (no. new cases, % confirmed cases) in Israel and in the patient’s town on the date of the exam and on the date the encounter record was created, closure indicator, mobility change in residential areas, COVID-19 wave indicator, number of days since Jan 1, 2019. For MG exams we extracted additional features: last MG BI-RADS score, last density, time to last MG exam, last US-breast BI-RADS, time to last US-breast exam, last MR-breast results, time to last MR-breast, last breast biopsy results, time to last breast biopsy.

### Development of risk models for clinical findings

Our models considered unique (patient, date) pairs. We randomly partitioned the set of patients into train (56%), validation (14%), and test (30%). We trained our prediction models on the train dataset and evaluated them on the validation dataset during the development process. The final models were evaluated on the held-out dataset. To enable the estimation of finding risks for all the patients in our study, we randomly partitioned the patients in the training set into two disjoint groups, and trained a prediction model on each of these groups. Therefore, for train patients we inferred the risk using the model on which they were not trained. For the remaining patients, we computed the average of the two models. The models only used features that were extracted from the time period before the exam. We considered the dates of CCI 2018 and 2020 to be 1.1.2019, and 1.1.2021 respectively, and used the latest one that occurred before the date of the exam.

We included the following set of features in our models: age, gender, socio-economic status, marital status, distance from exam facility, urgent findings in last 90 days, imaging procedures on that day, pre-exam urgency, wait time, weekend and holiday indicators, comorbidities, number of hospitalization in the last 90 days, MG specific variables (in case of MG), %confirmed cases measures, mobility change measures, and closure indicator.

The accuracy of the prediction models was evaluated with the area under the ROC curve (AUC). We used the DeLong [1] method to compute 95% confidence intervals for AUCs, and also to assess the significance of the difference between two AUCs that were computed on the same data.

## Additional results

### Predictors for clinical findings

To shed light on the conditions in which there was a greater risk for clinical findings, we trained and analyzed models to predict this risk in MG, CT, and MR exams. The AUCs of our prediction models were MG=0.75 [CI95: 0.74-0.76], CT=0.74 [CI95: 0.74-0.75], and MR=0.76 [CI95: 0.75-0.77]. Figure S1 presents the results of a SHAP analysis of these models, listing the top-20 most impactful features. In the MG model, two MG-specific features were found to be most impactful: “last MG BI-RADS”, which corresponded to the results in previous MG exams (with high BI-RADS indicating a clinical finding), and “last breast density”, which is a known risk factor for MG findings. Risk factors that were common to all models included: shorter wait time, previous findings, high pre-exam urgency, older age, male gender, lower socio-economic status, and recent higher utilization of healthcare services. Time-based features related to COVID-19 were also among the top-20 impactful features in all the three models: “residential mobility change”, which was high during closure periods, and “%confirmed cases” measures, which indicate COVID-19 morbidity. Removal of the time-based features from the training of our models significantly reduced the AUC for MR ($\Delta AUC=-0.02, P=3\times{10}^{-12}$) and CT ($\Delta AUC=-0.006, P=2\times{10}^{-5}$) models; for MG, the reduction was small and statistically insignificant ($\Delta AUC=-0.0004, P=.8$).

### Clinical findings during COVID-19

#### Musculoskeletal (MSK) findings

Following the discovery of the substantial rise in MR-MSK findings, we also examined CT-MSK finding rate. However, in oppose to MR-MSK, CT-MSK did not show any increase in the finding rate during COVID-19. A comparison of the characteristics of MR-MSK patient visit instances (n=24,923) with those of CT-MSK (n=11,063) revealed that the former were a younger age (mean ± standard deviation, 46±15 vs. 51±17 year), with fewer comorbidities (CCI 2020: 0.7±1.2 vs. 1±1.6), higher percentage of marital status single (53% vs. 47%), and higher socio economic status (SES: 7.0±1.7 vs. 6.8±1.7). Finally, MR-MSK wait times were much longer compared to CT-MSK (33±31 vs. 11±10).

#### Neuroimaging findings

During the covid period we observed a significant rise in the finding rate for CT-brain (6.2% vs. 4.9%, $P=2\times{10}^{-7}$) and MR-neuro (6.8% vs. 5.6%, $P=2\times{10}^{-6}$) exams. Our results also indicated an overall significant increase in the finding rate of MR-head, a subclass of MR-neuro, (8.3% vs. 7%, P=.0004). When focusing on waves 3 to 4, we observed a significant increase in the daily finding rate for CT-brain (7.7% vs. 4.9%, $P=3\times{10}^{-10}$) and MR-neuro (6.5% vs. 5.6%, $P=.005$, t test), but not for MR-head (8% vs. 7%, $P=.06$).

We compared CT-brain and MR-head patient visits, with 25,724 and 18,988 visit instances, respectively. Both were mostly (59-60%) women, with similar high levels of comorbidities (CCI 2020: 1.5±2 vs. 1.5±2.1), but CT-brain patients were older (57±18 vs. 52±17 years), with lower socioeconomic status (SES score 6.5 ± 1.7 vs. 6.9 ± 1.8), and had a lower rate of cancer (14% vs. 20%). As expected, CT-brain wait times were shorter than those of MR-head (15±13 vs. 49±49).

#### CT-chest findings

Overall, we did not observe any significant increase in the proportion of CT-chest exams with findings during the covid period (5.7% vs. 5.6%, $P=.3$, Table S5 in Multimedia Appendix 1, Figure 3e). However, a finer inspection revealed a significant decrease in CT-chest finding rate during the second wave (3.9%, $P=1\times{10}^{-5}$), followed by a significant increase during waves 3 to 4 (7.1%, $P=.003$).

#### Mammography (MG) findings

During the covid period, MG exams showed an increase in the finding rate: 3% vs. 2.8% in the pre-covid period ($P=.0009$, proportion test). An outstanding increase in the daily finding rate was observed during the first wave, along with an increase in the average predicted finding risk (Figure 3a). We observed a significant increase in the proportion of non-screening (aka diagnostic) exams during the covid period (SMD=0.45, $P=8\times{10}^{-16}$, Table S1 in Multimedia Appendix 1). In our dataset, the finding rate in non-screening MG exams was three times greater than that of MG screening exams (5.9% vs. 2%). During covid period, the finding rate in MG screening exams had shown a small, yet statistically significant, increase from 2% to 2.1% ($P=.03$, proportion test). The finding rate in non-screening exams had a slight increase, which was not found to be statistically significant (6% vs. 5.9%, $P=.3$, proportion test). Thus, the increase in MG finding rate can be attributed mostly to the increase in non-screening MG exams.

### References

1. DeLong ER, DeLong DM, Clarke-Pearson DL. Comparing the Areas under Two or More Correlated Receiver Operating Characteristic Curves: A Nonparametric Approach. Biometrics [Wiley, International Biometric Society]; 1988;44(3):837–845. [doi: 10.2307/2531595]


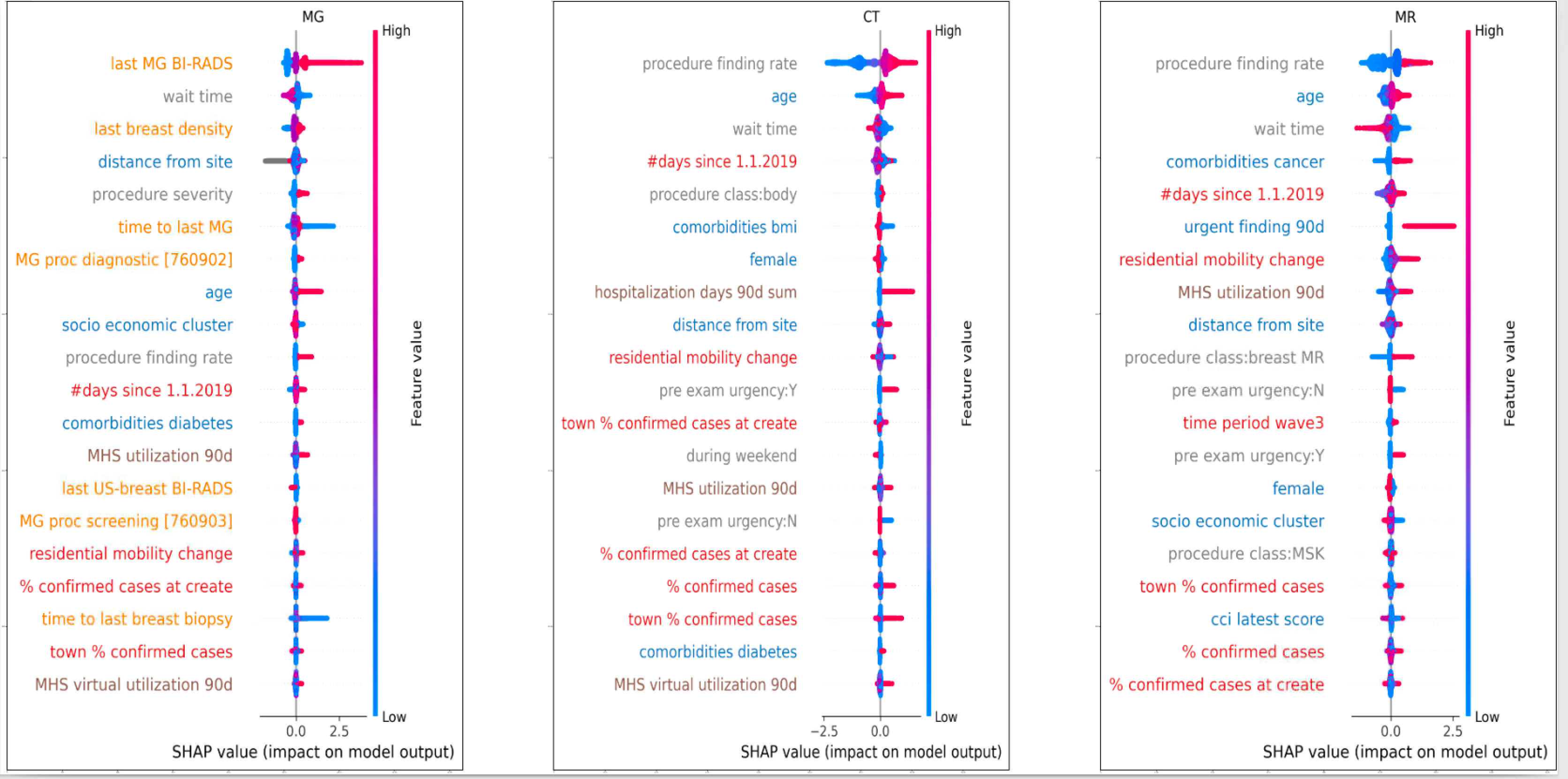


Figure S1. Risk factors for urgent findings in MG, CT and MR exams. Feature names are colored by group: grey=exam information; red=time dependent; orange=mammography (MG) history; blue=patient’s demographics and comorbidities; brown=90 history of healthcare utilization. Each point represents a single data sample (i.e., patient at a date). Point color indicates the corresponding feature value (red=high, blue=low, gray=missing); X-axis denotes the estimated effect of the feature on predicted finding risk for the sample, which can be positive (increasing predicted risk), or negative (decreasing predicted risk).

**
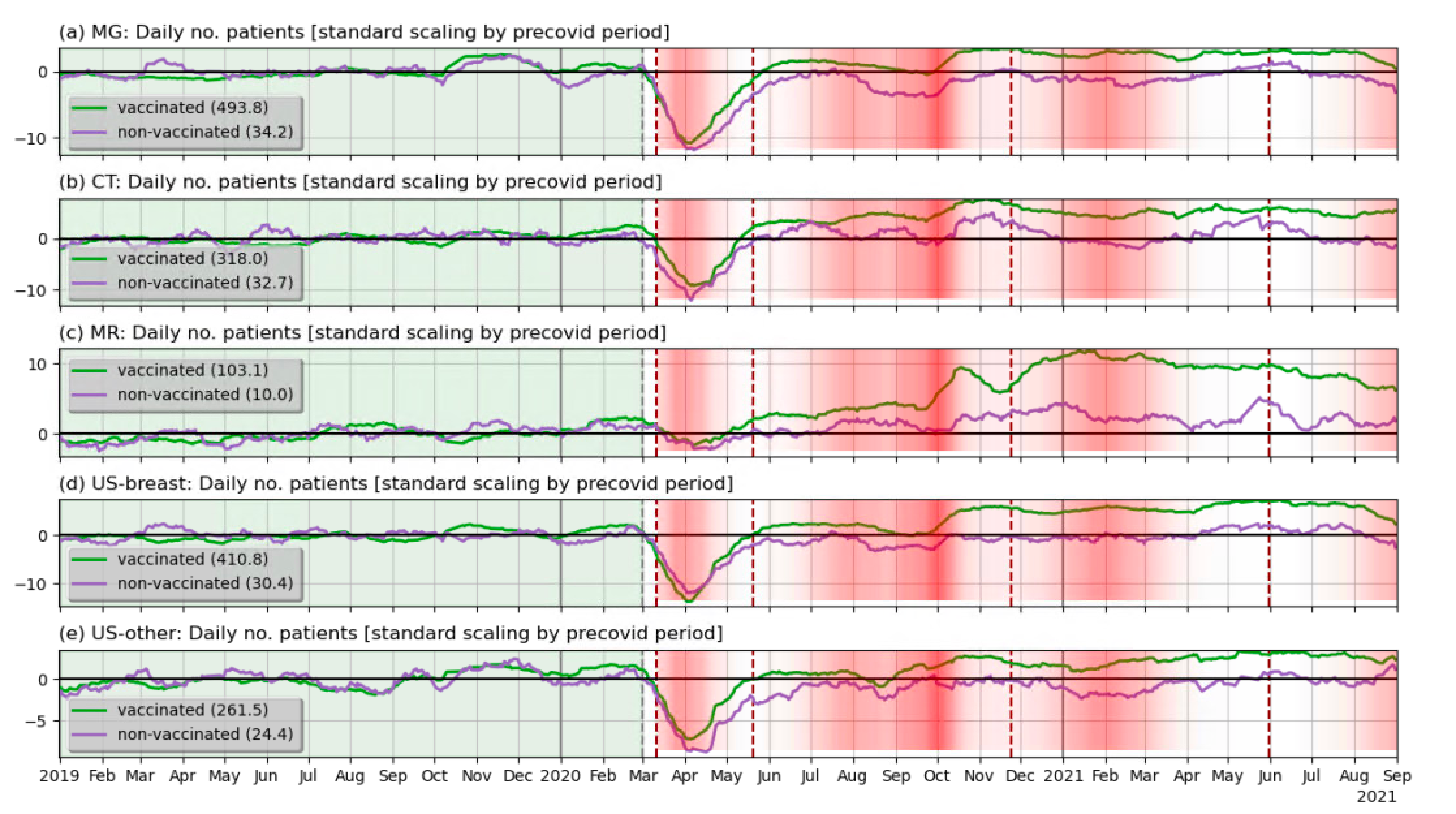
**

**Figure S2. History of medical imaging utilization: vaccinated vs. vaccine-hesitant.** All patients that received at least one COVID-19 vaccination by the end of the study were classified as “vaccinated”, while remaining patients were classified as “vaccine-hesitant”. Historical daily utilization measures of vaccinated vs. vaccine-hesitant groups are presented for each of the 5 most common modalities: (a) mammography [MG], (b) CT, (c) MR, (d) breast ultrasound [US-breast], and (e) other non-breast ultrasound [US-other]. All measures were computed with respect to the set of unique patients with exams on each date, normalized with standard scaling using the mean and standard deviation values computed during the pre-covid period, and smoothed by taking the average of a centered 28-day window. The scaling mean values appear in round brackets in the legends.

**
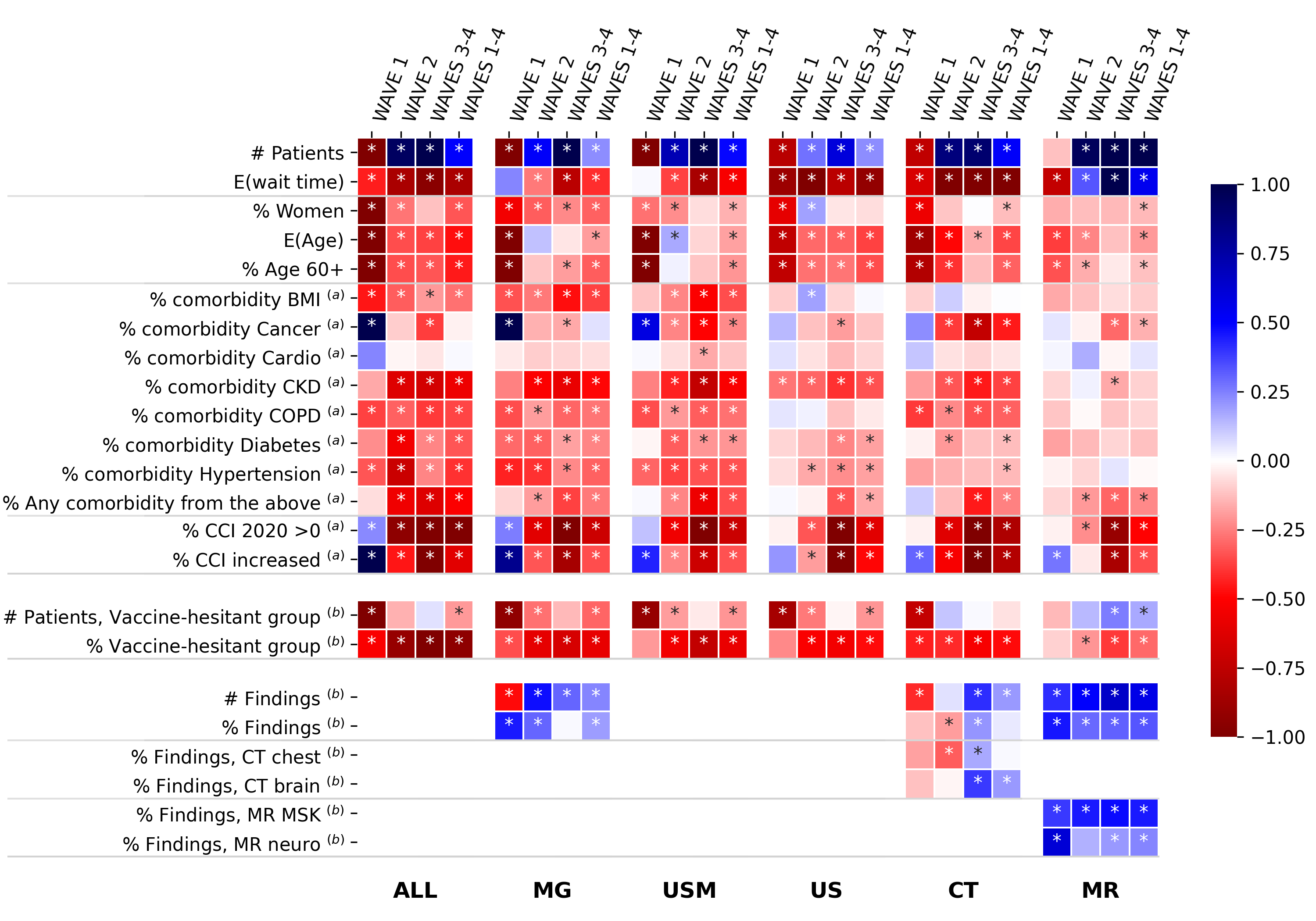
**

**Figure S3. Changes in daily measures of medical imaging utilization and abnormal findings.**^(a)^ after correction to age and gender with AdvBal. ^(b)^ after correction to changes in medical imaging utilization with AdvBal. Color indicates the change in the daily measure, calculated as the standard mean difference (SMD) w.r.t. the pre-covid period. Significant changes are marked in “*”. The underlying values appear in Tables S1, S3 and S5 in Multimedia Appendix 2.exams on each date, normalized with standard scaling using the mean and standard deviation values computed during the pre-covid period, and smoothed by taking the average of a centered 28-day window. The scaling mean values appear in round brackets in the legends.
